# Towards carotenoid biofortification in wheat: identification of *XAT-7A1*, a multicopy tandem gene responsible for carotenoid esterification in durum wheat

**DOI:** 10.1186/s12870-023-04431-4

**Published:** 2023-09-06

**Authors:** C Rodríguez-Suárez, MD Requena-Ramírez, D Hornero-Méndez, SG Atienza

**Affiliations:** 1 Institute for Sustainable Agriculture, CSIC, Avda, Menéndez Pidal s/n, E-14004 Córdoba, Spain; 2 Department of Food Phytochemistry, Instituto de la Grasa, CSIC. Campus Universidad Pablo de Olavide, Edificio 46. Ctra. de Utrera, Km 1, E-41013 Sevilla, Spain

**Keywords:** Carotenoids, Esterification, GDSL esterase/lipase, Tandem copies, Biofortification, Durum wheat

## Abstract

Yellow pigment content, mainly due to the accumulation of carotenoids, is a quality trait in durum wheat grain as it confers the bright yellow color to pasta preferred by consumers. Also, carotenoids are essential nutrients exerting important biological functions in human health. Consequently, biofortification strategies have been developed in many crops to increase carotenoid content. In this context, carotenoid esterification is emerging as a new breeding target for wheat biofortification, as carotenoid esters have been found to promote both carotenoid accumulation and stability. Until recently, no carotenoid esters have been identified in significant proportions in durum wheat grains, and interspecific breeding programs have been started to transfer esterification ability from common wheat and *Hordeum chilense*.

In this work, *XAT-7A1* is identified as the gene responsible for carotenoid esterification in durum wheat. Sequencing, copy number variation and mapping results show that *XAT-7A1* is organized as tandem or proximal GDSL esterase/lipase copies in chromosome 7A. Three *XAT-7A1* haplotypes are described: Type 1 copies, associated with high levels of carotenoid esters (diesters and monoesters) production and high expression in grain development; Type 2 copies, present in landraces with low levels of carotenoid esters (monoesters) or no esters; and Type 3 copies, without the signal peptide, resulting in zero-ester phenotypes.

The identification of *XAT-7A1* is a necessary step to make the carotenoid esterification ability available for durum and bread wheat breeding, which should be focused on the Type 1 *XAT-7A1* haplotype, which may be assessed as a single gene since *XAT-7A1* copies are inherited together.

**Supplementary Information:**

The online version contains supplementary material available at 10.1186/s12870-023-04431-4.

## Background

Durum wheat, *Triticum turgidum* L. subsp. *durum* (Desf.) is an important crop worldwide, with an annual production of 40 million tons [1] and with special relevance in the Mediterranean basin, due to its historical origin, distribution and cultural uses [2]. High yellow pigment content (YPC), mainly due to carotenoid accumulation, has been traditionally considered as a quality trait in durum wheat grain, as it confers the bright yellow color to pasta preferred by consumers. Lutein is the main carotenoid in the endosperm of *Triticum* species [3–5]. Carotenoids are lipophilic pigments synthesized by photosynthetic organisms and some fungi and bacteria. They play important roles in plants, contributing to photosynthesis as accessory light-harvesting pigments, preventing the formation of destructive chemical species, quenching the energy excess, and serving as antioxidants [6]. Additionally, they are essential nutrients with important biological functions in human and animal health, being associated with antioxidant and provitamin A activity, enhancement of the immune response, inhibition of carcinogenesis and reduction on the risk of developing cardiovascular and other degenerative or chronic diseases [7, 8]. Consequently, biofortification strategies have been developed in many crops [9] through transgenic means, such as the well-known Golden Rice [10], the Carolight® maize [11] and the tomato enriched for saffron apocarotenoids [12]; or by using natural variation and traditional breeding such as in vitamin A-biofortified maize [13], orange sweet potato or cassava (https://www.harvestplus.org/home/crops/).

Also in durum wheat, increasing YPC and carotenoid content has been a breeding target [14], which has been accompanied by an interest in deciphering the genetic basis of the trait. Carotenoid levels in plants are known to be the result of three interacting variables: biosynthetic rate, degradation rate and storage cell capacity [15]. As a result, abundant information is available about the genetic control of carotenoid synthesis and degradation in wheat [16–18]. Conversely, other factors contributing to carotenoid accumulation in wheat grains are not so well understood. In this context, carotenoid esterification is gaining relevance. Carotenoids can be in their free form or esterified with fatty acids. Esterification is known to enhance carotenoid sequestration in plants, providing a metabolic sink acting as a regulatory mechanism leading to higher carotenoid concentrations [19]. Also, carotenoid esters have shown to be more stable than non-esterified carotenoids [20] and to prevent carotenoid degradation during storage of wheat and tritordeum grain and flour [21–24]. Furthermore, lutein diesters appeared to be more resistant to lipoxygenase degradation than free and lutein monoesters in noodle sheets [25]. Thus, esterification is emerging as a new breeding target to improve both carotenoid accumulation and stability in durum wheat.

Carotenoid esters had been found in grains of hexaploid and diploid wheats and in tritordeum but not in tetraploid wheats [3–5] until the recent characterization of a collection of Spanish durum wheat landraces, which resulted in the identification of four accessions with a high presence of lutein esters (diesters and monoesters), and eleven accessions with lower contents of esters (monoesters only) [26]. The study of the genetic control of lutein esterification had been addressed in the D and H^ch^ genomes [27–29]. Further studies in common wheat identified a xanthophyll acyl transferase gene (XAT) belonging to the GDSL esterase/lipase (GELP) family located in chromosome 7D (*XAT-7D*) as responsible of lutein esterification in this cereal [30]. Shortly after, *XAT-7Hch*, orthologous of *XAT-7D*, was reported as the gene responsible of lutein esterification in *H. chilense* and tritordeum [31], suggesting a common origin of the mechanism for lutein esterification in the Triticeae species. Thus, carotenoid esterification ability found in durum wheat landraces may be expected to be mediated by the same mechanism, through an orthologue XAT gene in the tetraploid genome. Due to the special importance of carotenoids in durum wheat, where lutein content is a primary selection target for its correlation with pasta color, elucidating the genetic basis of carotenoid esterification in this species would be a milestone in durum wheat breeding. Due to this relevance, and prior to the identification of durum wheat sources [26], the transfer of *XAT-7D* and *XAT-7Hch* genes through interspecific crosses has been proposed and attempted, respectively [32, 33]. However, the availability of durum wheat donors with carotenoid esterification ability eliminates the difficulties associated with interspecific breeding, although both strategies may be complementary.

Thus, the aim of this work is to identify the gene responsible for lutein esterification in durum wheat. For this purpose, several methodologies have been used for answering the questions that arose during the investigation. In this sense, genomic sequencing, expression analysis, copy number variation and genetic and QTL mapping strategies were successfully combined to achieve the objective set forth in this work.

## Results

### Candidate genes for carotenoid esterification in durum wheat

A common genetic mechanism for carotenoid esterification has been proposed for Triticeae species [29, 31]. Thus, the starting point for identifying the gene responsible for carotenoid ester production in durum wheat grains was that the same mechanism mediated by GDSL esterase/lipase genes described in bread wheat [30] and in *H. chilense* genomes [31], also operated in durum wheat. The hypothesis was that an orthologous and functional copy of *XAT-7D*, the gene responsible for carotenoid esterification in common wheat [30], might be present in the durum wheat esterifying genotypes. We searched for the orthologous genes of *XAT-7D* (TraesCS7D02G094000) in the available tetraploid genomes: *T. turgidum* (Svevo.v1) and *T. dicoccoides* (WEWseq_v.1.0) accessible at EnsemblPlants (https://plants.ensembl.org/). The only gene assigned as an orthologue in durum wheat was TRITD4Av1G231510, which had the same exon-intron structure of *XAT-7D* but shared a sequence identity below 73%. In addition, we performed a BLASTn search of *XAT-7D* against the durum wheat genomes to identify other putative candidates. Two more genes, TRITD4Av1G231520 and TRITD4Av1G231670, showed similar identity and coverage values with *XAT-7D* that TRITD4Av1G231510. Additionally, TRITD4Av1G231840 in *T. turgidum* and TRIDC4AG057350 in *T. dicoccoides* were identified. Both genes were truncated forms compared to *XAT-7D*, as they only shared the first exons and introns. Conversely, they were around 95% identical in the region in common. The results of this search are summarized in Additional file 1. None of the genes identified in the two available durum wheat genomes matched what was expected for full-length functional orthologous copy of *XAT-7D*.

Thus, several primer pairs were designed based on the 5’ and 3’ regions of TraesCS7D02G094000 and its homoeologue TraesCS4A02G397900, considering also the sequences at 5’ of genes TRITD4Av1G231840 and TRIDC4AG057350 in order to tag a putative complete and functional copy in durum wheat. The primer pair XAT_dw-Fw / XAT_dw-Rv (Additional file 2) amplified a PCR fragment expected for a complete copy (1.5 Kb approximately) in some genotypes from a durum wheat Spanish landraces collection, previously characterized for their grain carotenoid and xanthophyll esters profile [26].

### Characterization of XAT-like sequences in durum wheat

Out of the 156 durum wheat landraces tested, 28 lines showed amplification for XAT_dw-Fw / XAT_dw-Rv (Additional file 3). All durum wheat lines from the collection producing carotenoid esters showed amplification: the four genotypes producing both diesters and monoesters in significant proportion and the eleven genotypes producing only monoesters in small amounts described in [26]. Additionally, thirteen lines showing no esters in their carotenoid profile showed positive amplification. Accessions from the three phenotypes were selected for cloning and sequencing of the amplified fragment: BGE047520 and BGE047535 producing diesters and monoesters, BGE048494 producing only monoesters and BGE047499 with no esters. The variety ‘Athoris’ was also included as it was the zero-ester parental of the population developed for mapping purposes (see below).

In all cases, a 1,564 bp fragment was successfully sequenced. By comparing all the sequences obtained, they could be classified into three types which were designed as Type 1, Type 2 and Type 3 considering exclusive single nucleotide polymorphism (SNP) positions (Table 1). Type 1 sequences were identified only in the two genotypes producing significant levels of esters, both diesters and monoesters (BGE047520 and BGE047535). Type 2 was the form present in BGE048494, the genotype which produces low amounts of esters (only monoesters), and Type 3 was exclusive to the genotypes that were not able to produce esters BGE047499 and ‘Athoris’. Table 1 summarizes the polymorphic sites among sequence types as well as the expected amino acid substitutions in the predicted proteins.

**Table 1:** Polymorphic sites in XAT- like durum wheat sequences identified and the amino acid substitutions expected in the predicted proteins. The exon- intron structure was determined by comparison with *XAT-7D* gene of common wheat

		XAT-like sequence type										
Location	bp	1.1	1.2	1.3	1.4	1.5	1.6	1.7	2	3.1	3.2	Substitution pos^a^
5' UTR	24	C	C	-	C	C	C	C	G	C	C	-
1st ATG	36	T	T	T	T	T	T	T	T	C	C	T (M)- C (no ATG)
intron1	333	A	G	G	A	G	G	A	G	G	G	
exon2	400	A	A	A	A	A	A	A	G	A	A	synonymous
intron2	504	G	G	G	G	G	G	G	A	G	G	
exon3	573	A	A	A	A	A	A	A	G	A	A	synonymous
exon3	644	T	C	C	C	C	T	C	C	C	C	synonymous
exon3	691	G	G	A	A	A	G	G	G	G	G	166 G (G)- A (D)
exon3	705	G	G	G	G	G	G	G	G	T	G	171 G (V)- T (F)
exon3	750	G	A	G	G	G	G	A	G	G	G	186 G (A)- A (T)
exon3	777	G	G	G	G	G	G	G	G	A	A	195 G (G)- A (R)
exon3	788	C	C	A	A	C	C	C	C	C	C	198 C (N)- A (K)
intron3	857	T	T	T	T	T	T	T	C	T	T	
intron4	1180	A	A	A	A	A	A	A	T	T	T	
intron4	1193	T	T	T	T	T	T	T	C	T	T	
intron4	1203	A	A	A	A	A	A	A	T	A	A	
intron4	1220^b^	A	A	A	T	T	T	A	A	A	A	
intron4	1236	G	G	G	G	G	G	G	T	G	G	
intron4	1243^b^	T	T	T	T	T	T	T	T	C	C	
intron4	1270	G	G	G	A	A	A	G	G	G	G	
exon5	1458	G	G	G	T	T	T	G	G	G	G	328 G (W)- T (C)
exon5	1490	T	T	T	T	T	T	T	C	T	T	339 T (V)- C (A)
	Clones^c^	3/5	5/2	1/1	1/1^d^	0/1	1/0	1/0	5	2/1	3/3	

^a^Amino acid substitution and position in the expected protein

^b^Polymorphisms tagged with markers SNP_1220 and SNP_1243 for mapping purposes

^c^Number of clones assigned to types: for Type 1 sequences, first number corresponds to BGE047535 clones and second number to BGE047520 clones; Type 2 sequences are all from BGE048494; for Type 3 sequences, first number corresponds to ‘Athoris’ clones and second number to BGE047499 clones

^d^In BGE047535: C at 510 bp + T at 1500 bp; in BGE047520: T at 510 bp + C at 1500 bp

Two alternative forms at position 705 (T/G) were consistently identified in Type 3 sequences in both genotypes BGE047499 and ‘Athoris’, which were named Type 3.1 and Type 3.2. In Types 2, 3.1 and 3.2 sequences other SNPs were detected in only one clone and, as polymerase amplification or sequencing errors could not be excluded, they were not identified as new subtypes.

Type 1-like sequences revealed a surprising diversity resulting from different combinations of alternative bases at positions 333 (A/G) at intron 1; 644 (T/C), 691 (A/G), 750 (A/G) and 788 (C/A) at exon 3; 1220 (A/T) and 1270 (G/A) at intron 4 and at 1458 (G/T) in exon 5 (Table 1). Types 1.1, 1.2, 1.3 and 1.4 copies were identified in both BGE047520 and BGE047535, being 1.1 and 1.2 the most represented copies (Table 1). Type 1.3 sequences were 1,536 bp – length, as they were 25 bp shorter in the 5’ UTR. Additionally, other two different combinations designed as 1.6 and 1.7 were found in BGE047535, as well as another one in BGE047520 named 1.5. Other SNPs were detected in these sequences and in other clones analyzed but, only those consistent (i.e alternative bases at the positions formerly described were identified in more than one sequence), were considered to define different Type 1 copies. Thus, the existence of more subtypes within this class cannot be ruled out.

Additionally, a small sample of clones from other landraces showing XAT amplification were sequenced in order to identify copy types. Due to the high diversity of XAT sequences shown above, they were only assigned to Type 1, 2 or 3 and were not analyzed at the subtype level. BGE047507 and BGE047536 harbor Type 1 sequences; BGE012301, BGE045645, BGE048496, BGE047498 and BGE047503 Type 2, and BGE045628, BGE045676 and BGE048499 Type 3 (Additional file 3).

All sequences shared about 96.5 % identity with TraesCS7D02G094000 and none of them were represented in the reference genomes of *T. turgidum* (Svevo.v1) or *T. dicoccoides* accession 'Zavitan' (WEW_v.1.0) in the Ensembl repository. Conversely, Type 3.2 sequence was 100% identical to gene LOC119344677 available from the new version of *T. dicoccoides* genome (WEW_v.2.0) at the NCBI, although its chromosomal location has not been established.

The exon-intron structure was estimated by comparing with TraesCS7D02G094000 and the expected proteins were predicted. A 22 amino acid-length signal peptide (cleavage site between positions 21 and 22) was detected in Type 1 and Type 2 sequences predicting an extracellular protein location, and both types encoding for 351 amino acid length peptides (Additional file 4). However, the signal peptide was lacking in Type 3 sequences due to the T / C substitution at position 36 (Table 1) interrupting the ATG start codon and giving rise a to shorter protein of 320 amino acids. All sequences belong to the GELP family and conserve the catalytic triad consisting of the S in block I domain and the D and H residues in block V [34] (Additional file 4).

### XAT-like sequences copy number variation

To further investigate the nature of the great diversity found for XAT-like sequences in durum wheat, a copy number analysis was carried out by qPCR. Amplification of a 131 bp fragment identical in all Type 1, 2 and 3 copies was quantified in BGE047535, BGE047520, BGE048494, BGE047499 and ‘Athoris’. The landrace BGE018318, without XAT copies, was included as negative control. The lowest copy number was detected in BGE048494 with 2.07 copies. In the same range of variation were BGE047535, BGE047520 and ‘Athoris' with 5.54, 5.26 and 5.73 copies, respectively. The landrace BGE047499 showed the highest number of copies with an estimation of 8.68. No copies (0.12) were detected in the landrace BGE018318 used as negative control (Table 2).

**Table 2:** XAT-like sequences copy number (ratios) per haploid genome of selected genotypes and individuals from the BGE047535 × ‘Athoris’ F_2_ population with parental and heterozygous haplotypes (hap)

	Ratios						
	XAT/Ref1	XAT/Ref2	Ref1/Ref2	Mean XAT^a^	SD^b^	Upper limit^c^	Low limit^d^
BGE047535	5.48	5.60	0.98	5.54	1.04	5.74	5.34
BGE047520	5.19	5.32	0.98	5.26	1.12	5.89	4.69
BGE048494	2.10	2.05	1.03	2.07	1.21	2.51	1.71
BGE047499	8.67	8.70	1.00	8.68	1.21	10.50	7.18
‘Athoris’	5.91	5.56	1.06	5.73	1.08	6.16	5.34
BGE018318	0.12	0.12	1.04	0.12	1.45	0.18	0.08
F_2_ BGE047535 hap	6.00	5.39	1,16	5.68	1.15	6.56	4.93
F_2_ ‘Athoris’ hap	5.30	6.60	0.80	5.91	1.10	6.53	5.36
F_2_ heterozygous hap	6.00	6.40	0.91	6.19	1.14	7.08	5.42

^a^Mean XAT: Geometric mean of XAT/Ref1 (TaFAD6) and XAT/Ref2 (RLIa) ratios

^b^SD: geometric standard deviation

^c^Upper limit= (Mean XAT x SD) + Mean XAT

^d^Lower limit= (Mean XAT / SD) + Mean XAT

Additionally, seven F_2_ individuals with different phenotypic profiles and characterized as BGE047535, ‘Athoris’ or heterozygous haplotypes (based on the SNP_1220 and SNP_1243 genotyping; see below in *Genetic mapping of esterification ability*), were also tested for copy number variation. The average number of copies for F_2_ BGE047535, ‘Athoris’ and heterozygous haplotypes were 5.68, 5.91 and 6.19 copies, respectively, in the same range of variation that the parents of the mapping population (Table 2).

These results show that XAT-like sequences are present in more than one copy in all the genotypes analyzed, accounting for the diversity detected by sequencing. Additionally, copy number is variable among them, ranging from 2.07 to 8.68 copies in the genotypes analyzed.

### Transcript profiling of XAT-like sequences during grain development

An expression analysis was carried out in three distinct phenotypic classes considering the carotenoid esters profile, and whose XAT-like copies have been sequenced: two genotypes (BGE047535 and BGE047520) with a relevant presence of lutein esters (both mono- and diesters), one genotype (BGE048494) with lower content of esters (only monoesters) and one genotype (BGE047499) with no esters, corresponding to the defined sequence Types 1, 2 and 3, respectively. The primer pair for RT-qPCR was designed at the end of exon 4 and the beginning of exon 5, where no polymorphisms were detected among all sequences, to avoid a preferential amplification of any copy. Grain samples from three developing stages (St) were used for transcript profiling: St1 (Zadoks 77, 18 dpa), St2 (Zadoks 83-85, 25 dpa) and St3 (Zadoks 87, 30 dpa), corresponding to the caryopsis late milk, early-soft dough and hard dough developmental stages, respectively.

**Fig. 1:**
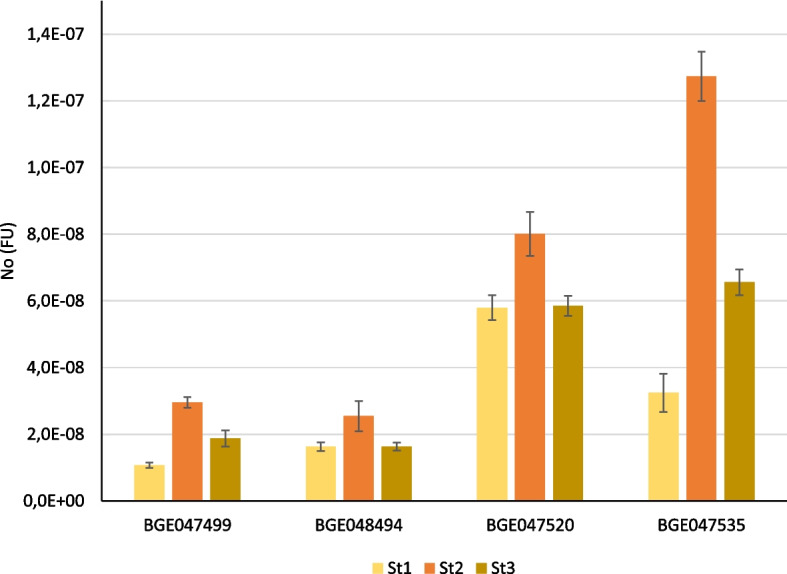
Transcript profiling of XAT-like sequences in developing grains of selected durum wheat genotypes. BGE047520 and BGE047535 show high proportions of carotenoid esters, both diesters and monoesters; BGE048494 show low levels of carotenoid esters, only monoesters; and BGE047499 shows no esters. Starting concentration of the amplicon (N0) in fluorescence units (FU) is normalized to the geometric mean of three reference genes. St1: late milk; St2: early-soft dough; St3: hard dough

A similar transcriptional profile was observed in the four genotypes (Fig. 1), with maximum expression at St2 (early-soft dough). Conversely, expression values were dramatically higher in the landraces BGE047535 and BGE047520, producing carotenoid monoesters and diesters in a high percentage, than in the other two genotypes producing only monoesters in a low percentage or no esters (BGE048494 and BGE047499, respectively).

### Genetic mapping of esterification ability

**Fig. 2:**
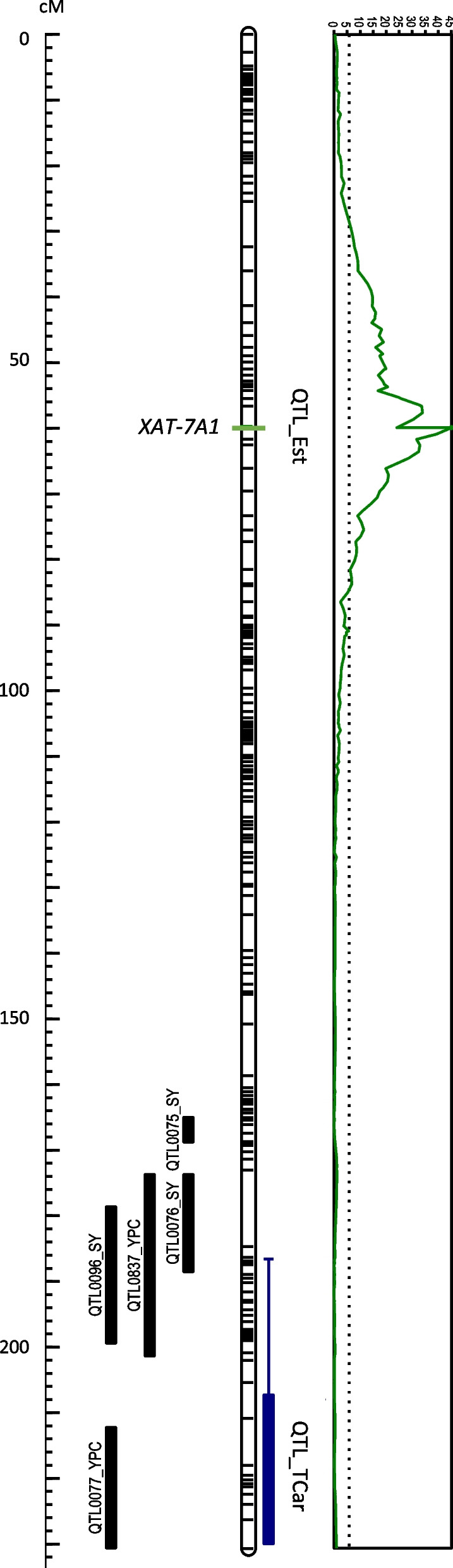
Genetic map of chromosome 7A. Genetic map of 7A chromosome (genetic distance in cM is shown at left) and QTL mapping of total carotenoid content (QTL_TCar) and carotenoid esterification (QTL_Est). QTL_TCar (blue bar) co-locates with QTLs described in previous works for semolina color and grain yellow pigment content (YPC) [17, 36, 37]. QTL_Est co-locates with *XAT-7A1* (mapped through SNP_1243 and SNP_1220 markers) at the maximum LOD (LOD profile shown in green, right)

An F_2_ population derived from the cross between the esterifying genotype BGE047535 and the zero-ester variety ‘Athoris’ was constructed for gene mapping purposes and grown under field conditions. Carotenoid profile was evaluated in the parental lines and in the progeny consisting of 120 individuals. A subset of 90 F_2_ plants and the parent lines were genotyped by using DArTseq markers, which generated the data set for the linkage map construction spanning 2,277.4 cM (Additional file 5). The detailed carotenoid content and profile (including esterification) of the grain harvested in each F_2_ plant is shown in Additional file 6. Lutein was the major carotenoid followed by zeaxanthin and minor amounts of α- and β-carotene. As expected, the population segregated for esterification ability. The observed segregation for carotenoid esters presence/ absence showed a good fit to the expected ratio for a single dominant gene (χ^2^ = 1.11; *p* = 0.292). Total carotenoid content and degree of esterification (%) were used for QTL identification. A QTL for esterification ability (QTL_Est) was detected on chromosome 7A explaining 90% of the phenotypic variation. In addition, a QTL for total carotenoid content (QTL_TCar) was detected in the distal part of chromosome 7A explaining 24% of the total variation (Table 3, Fig. 2).

**Table 3:** QTLs for total carotenoid content (QTL_TCar) and esterification ability (QTL_Est) in the F_2_ population derived from the cross BGE047535 × ‘Athoris’

QTL	LOD	Peak LOD position	Chromosome	Additive	Dominance	R2	*P*^a^
QTL_Est	45.1	60.7	7A	6.96	0.76	90	0.000
QTL_TCar	7.4	212.1	7A	-0.39	-0.08	24	0.000

^a^The significance (*P*) was calculated considering the permutation test

A diagnostic marker was designed to detect the SNP at position 1243 bp between BGE047535 and ‘Athoris’ XAT-like copies (Table 1). All Type 1 variants detected in the parental line BGE047535 harbor a T at this position, whereas the two Type 3 variants identified in the parental line ‘Athoris’ harbor a C (Table 1). The SNP was scored in the total mapping population (Additional file 6) and showed a good fit to the 1:2:1 ratio expected for a single co-dominant locus (χ^2^ = 1.2; *p* = 0.545). The SNP_1243 marker mapped to chromosome 7A (60.7 cM) and co-localized with QTL_Est, indicating that XAT-like sequences are responsible for carotenoid esterification in durum wheat and thus this locus was named *XAT-7A1*.

A second SNP marker was designed to locate different copies of the BGE047535 haplotype in the mapping population. Type 1.4 and 1.6 copies harbor a T at position 1220 bp, whereas the remaining copies identified in BGE047535 and in the other parental line ‘Athoris’ harbor an A (Table 1). The two specific fragments of 357 bp and 457 bp (corresponding to the ‘A’ and ‘T’ copies, respectively) amplified in BGE047535 and, only the 357 bp fragment (‘A’-copies) was obtained in ‘Athoris’ as expected. The SNP_1220 marker was scored in the F_2_ mapping population (Additional file 6), where only two phenotypes were identified: A+T (homozygous for BGE047535 or heterozygous) and A (homozygous for ‘Athoris’), indicating that BGE047535 copies were inherited together or they were tightly linked. The segregation showed a good fit to the 3:1 ratio expected for a dominant marker (χ^2^ = 0.41; *p* = 0.52), co-segregated with SNP_1243 and then, co-located with QTL_Est for carotenoid esters content.

**Fig. 3:**
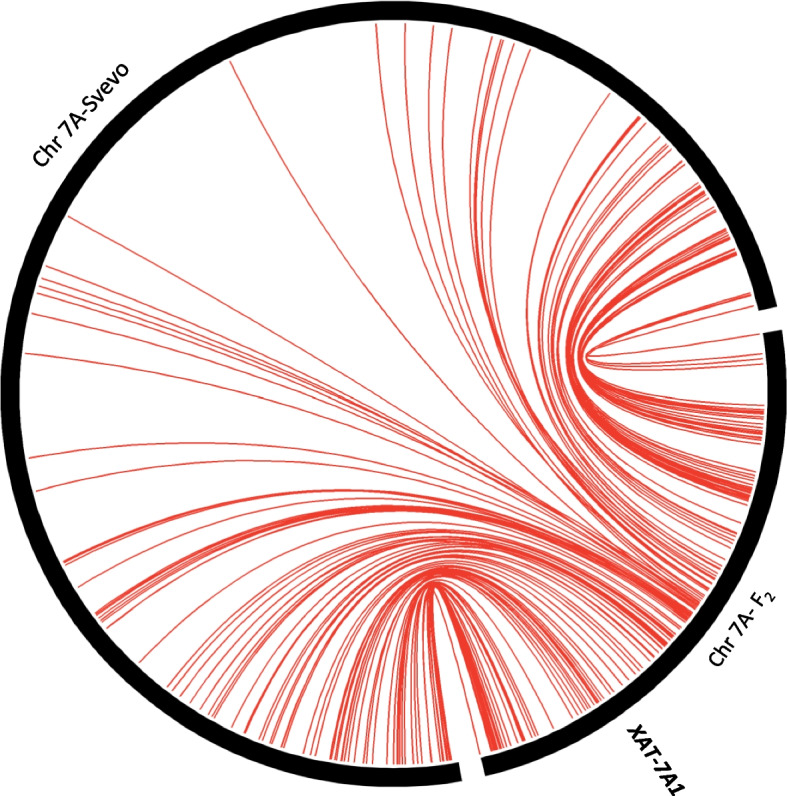
Collinearity between chromosome 7A. Collinearity between 7A chromosome (genetic *vs.* physical). Chromosome 7A map length in the mapping population BGE047535 × 'Athoris' is 233.657 cM and ‘Svevo’ chromosome 7A length is 728 Mbp. The expected physical position of *XAT-7A1* approximately corresponds to 54.2 Mbp

Figure 3 shows the collinearity between chromosome 7A (genetic *vs.* physical). The expected physical position of *XAT-7A1* would approximately correspond to 54,2 Mbp.

## Discussion

In this work, the gene responsible for carotenoid esterification in durum wheat is identified and named *XAT-7A1*. A candidate gene approach based on *XAT-7D*, the gene underlying carotenoid esterification in bread wheat [30], revealed that orthologous copies were present in some durum wheat lines from a Spanish landrace collection. Sequences of *XAT-7A1* gene were obtained from fifteen genotypes (fourteen landraces and ‘Athoris’) with different carotenoid esterification phenotypes. All sequences shared a high identity with *XAT* genes previously described in Triticeae as being responsible for carotenoid esterification (84 % with *XAT-7Hch*; 96.5 % with *XAT-7D*) and contain the conserved GDSL motif near the N- terminal [35] and the catalytic triad consisting of the active-site S in block I and the D and H amino acids in block V [34].

Within the landraces harboring *XAT-7A1* copies, different esterification phenotypes were observed. In the genotypes analyzed in this work, Type 1 sequences are present in the four accessions (BGE047507, BGE047520, BGE047535 and BGE047536; Additional file 3) producing carotenoid esters (diesters and monoesters) at a remarkable high percentage. Type 2 sequences can be associated with those accessions presenting low levels of carotenoid esters, mostly monoesters, (BGE012301, BGE045645 and BGE048496) or no esters (BGE047498 and BGE047503). In this case, due to the low amounts of monoesters shown, it is technically difficult to discern between monoester traces or no esters at all, which could explain both phenotypes found in accessions with Type 2 *XAT-7A1* sequences.

Type 3 sequences are characteristic of the genotypes with no esterification ability such as BGE045628, BGE045676, BGE047499 and BGE048499 (Additional file 3). These type 3 *XAT-7A1* copies are lacking the signal peptide sequence directing the protein to the apoplastic region, where carotenoid esterification has been suggested to occur in common wheat grain [30]. An inappropriate location may explain the absence of enzymatic reaction, regardless of protein functionality.

Furthermore, a surprising variability of *XAT-7A1* sequences was detected within each genotype, which could not be explained by the hypothesis of two alternative alleles at a single locus. In our analyses, subtypes were only considered if a sequence variant was detected more than once, or if the polymorphic positions were consistent between them (Table 1). This conservative criterion underestimated the number of different *XAT-7A1* copies, as shown by copy number analysis carried out afterwards. The *XAT-7A1* gene was detected in more than one copy in all the genotypes analyzed, and the copy number was variable among them, ranging from 2.07 to 8.68 copies per haploid genome.

To locate the gene responsible for carotenoid esterification in durum wheat, a mapping population was developed by crossing the landrace BGE047535 and the zero-ester variety ‘Athoris’. The carotenoid ester profile was phenotyped in the F_2_ progeny, showing that the carotenoid esters presence/absence fit well with a single dominant gene segregation. The QTL analysis allowed the location of the carotenoid esterification ability in the short arm of chromosome 7A. The QTL_Est explained 90% of variation, indicating that carotenoid esterification in durum wheat was controlled by a single locus. Two SNP markers, SNP_1243 and SNP_1220, were designed in *XAT-7A1* sequences and scored in the F_2_ population, also showing segregations expected for single dominant loci. The co-localization of SNP_1243, polymorphic between BGE047535 and ‘Athoris’ copies (Table 1), with QTL_Est demonstrated that *XAT-7A1* was responsible for carotenoid esterification in durum wheat (Fig. 2). Marker SNP_1220, targeting a different polymorphism among BGE047535 copies (Table 1), co-segregated both with QTL_Est and with marker SNP_1243, showing that *XAT-7A1* copies were inherited together and suggesting a tandem organization. This fits with the hypothesis that *XAT-7A1* is actually a multicopy locus of linked copies rather than a single copy gene. The results of copy number analysis support this model, as all genotypes tested harbor more than one copy. Specifically, BGE047535 and ‘Athoris’ harbor between five and six *XAT-7A1* copies (Table 2). The same range of variation was observed in the F_2_ individuals analyzed, showing that *XAT-7A1* is located in the same chromosomal region (chromosome 7AS) in both parents and also that *XAT-7A1* copies segregate as a block, as expected for a multicopy tandem locus and in agreement with the SNP genotyping results discussed above.

Additionally, a QTL for total carotenoid content (QTL_TCar) was identified in the distal part of chromosome 7A (Fig. 2), co-locating with QTLs described in other works for semolina color and YPC [17, 36, 37] and in agreement with previous knowledge of grain pigment content inheritance in durum wheat [16].

To date, the two genes identified as responsible for carotenoid esterification in cereal grains [30, 31], and *XAT-7A1* described herein, belong to the GELP family. This protein family has been extensively studied in many plant species due to its important roles in pathogen defense, growth and development and stress responses [38]. At genomic level, it has been demonstrated that GELP genes are abundant and not evenly distributed on chromosomes, as shown in soybean [39], tomato [40] or rice [41]. It has also been proposed that segmental duplication and differences in evolutionary rates are the main causes of the increase in the number and diversity of GELP genes in cotton, resulting in gene and functional diversity [42]. Furthermore, in a recent genome-wide analysis of GELP gene family in bread wheat [43], three genes, *TaGELP452*, *TaGELP454* and *TaGELP455* were mapped to the distal part of the short arm of chromosome 7D, co-locating with *XAT-7D* (*TaGELP453*). Then, the model proposed for *XAT-7A1* as tandem copies of GELP genes is coherent with the genomic organization of this gene family described in other plant species, although the results presented herein do not make possible to distinguish between tandem or proximal copy duplications.

Transcriptional analysis results are also in agreement with *XAT-7A1* being responsible for carotenoid esterification in durum wheat. Expression is detected during grain development, with maximum values at 25 dpa (early-soft dough). Up-regulation was also reported at the same physiological stage for the *XAT-7D* and *XAT-7Hch* genes [30, 31]. For *XAT-7D* gene, transcript levels decrease from this stage on through ripening as happens with *XAT-7A1* expression (Fig. 1). *XAT-7A1* copies in the landraces BGE047535 and BGE047520, with Type 1 copies and producing carotenoid esters (diesters and monoesters), are highly expressed during grain development. BGE048494 and BGE047499 share the same trend and low expression levels. In the case of BGE048494, with Type 2 sequences, low expression levels correlate to the scarce presence of carotenoid esters, which are detected in a low percentage compared to BGE047535 and BGE047520. Regarding BGE047499, although expression is detected during grain development, the absence of carotenoid esters in this landrace (an in ‘Athoris’ also with Type 3 *XAT-7A1* sequences) seems to be related to the lack of signal peptide directing the protein to the apoplastic region, where carotenoid esterification has been suggested to occur in wheat grain [30]. Landraces with the maximum copy number (BGE047499 with 8.68) and the minimum (BGE048494 with 2.07) share low expression levels all through grain development. Therefore, expression levels are not associated with gene copy number but with gene copy type, as the highly expressed Type 1 haplotypes showed higher carotenoid esterification activity (Additional file 3). However, it cannot be excluded that an effect on expression and on phenotype is related to the number of copies within the Type 1 sequences, since the BGE047535 and BGE047520 genotypes used in this study had the same number of copies (Table 2).

In the landraces studied in this work, all *XAT-7A1* copies identified within a genotype belong to the same sequence type. In the case of Type 1 sequences, where more subtypes were detected, the possibility cannot be excluded of different functionalities among copies. However, for practical purposes, the Type 1 *XAT-7A1* copies have shown to be inherited as a block. Thus, transferring the esterifying ability through a breeding program may be assessed as for a single-gene strategy. The availability of durum wheat XAT donor sources facilitates intraspecific introgression into elite durum wheat lines. Nevertheless, the value of interspecific *XAT* sources for durum wheat breeding should be evaluated, such as the use of *XAT-7Hch* [32] or *XAT-7D* [30], since new substrate specificities or preferences may be added to the carotenoid esterification durum wheat catalog. Indeed, a significant effect on the carotenoid stability due to the fatty acid involved and the acylation position in the lutein molecule has been reported [44]. Moreover, *XAT-7A1*, located in chromosome 7A, could be considered for common wheat biofortification by pyramiding with the *XAT-7D* gene.

None of the *XAT-7A1* sequences identified in this work were present in the two available tetraploid wheat reference genomes [45, 46]. The lack of representation of *XAT-7A1* is not surprising and may be explained by its low frequency in durum wheat. Even in the landrace collection, which by definition is a diversity reservoir, *XAT-7A1* was only detected in the 18 % of the accessions. This is also consistent with the lack of previous reports of durum wheat accessions producing carotenoid esters in a significant proportion, especially diesters, until the characterization of the Spanish durum wheat landraces by [26]. Although reference genomes are valuable and essential resources, they do not fully capture intraspecific genomic variation. Thus, pangenomes are needed for a global genomic perspective of variation within a species. Single-nucleotide polymorphisms (SNPs), insertions or deletions (indels), presence/absence variation (PAV) and gene copy number variation (CNV) are sources of genomic variation known to influence agronomic traits. Pangenome studies in bread wheat have revealed that approximately 12% of genes have PAVs and 26% of genes were found in tandem duplications, indicating that CNV is a major contributor to genetic variation [47]. Graph pangenomes, such as the recently published Panache visualization tool for PAVs in bread wheat [48] are valuable resources to inspect intraspecific genomic variation. Large-scale structural variations have been also identified through bread wheat pangenome studies. For instance, a translocation between chromosomes 5B and 7B was detected in 66% of the 538 common wheat lines analyzed [47, 49]. Unfortunately, a durum wheat pangenome is still in progress. PAV in durum wheat short arm of chromosome 7A may be an explanation for the variability found for *XAT-7A1* in the collection. Also, a 4AL/7AS translocation or duplication may be responsible of this variation. Candidate genes initially identified by their identity with *XAT-7D* (Additional file 1) are all located in a region of the long arm of chromosome 4A between 646,988,559 and 647,955,659 Mbp in ‘Svevo’ genome, being also TRIDC4AG057350 in an equivalent position in ‘Zavitan’ genome (Additional file 1). Conversely, in this work, *XAT-7A1* is located in the short arm of chromosome 7A. The construction of a high-density consensus map of durum wheat combining segregation data from six mapping populations [50], suggested that a translocation event took place between 4AL and 7AS chromosomes considering discordant marker positions. Furthermore, evidences of a large translocation involving the same regions are also reported by [46] by comparing the ‘Svevo’ and ‘Zavitan’ genomes and through a survey of the genetic diversity of a Global Tetraploid Wheat Collection consisting of 1,856 accessions. Although this hypothesis is plausible, it needs further investigation.

## Conclusions

*XAT-7A1* is identified as responsible for carotenoid esterification in durum wheat and it is located in the short arm of chromosome 7A. All genotypes producing carotenoid esters harbor *XAT-7A1* gene copies, orthologous to *XAT-7D* and *XAT-7Hch* genes responsible for lutein esterification in common wheat and in *H. chilense* and tritordeum, respectively. *XAT-7A1* Type 1 copies are related to relevant production of carotenoid diesters and monoesters and to high levels of expression during grain development. Type 2 copies are present in landraces producing small amounts of carotenoid esters (mostly monoesters) or no esters, whereas Type 3 *XAT-7A1* copies, without signal peptide, results in zero ester production as happens in genotypes lacking this gene. Type 2 and 3 copies are both expressed at low levels during grain development. The majority of the durum wheat landraces do not harbor *XAT-7A1* and do not show esters in the carotenoid profile of mature grains. Sequencing, CNV and mapping results revealed that *XAT-7A1* is organized as tandem or proximal copy duplications and that all sequences within a genotype belong to the same type class. Also, *XAT-7A1* expression seems not to be associated to copy number but to gene copy type which is, at the same time, associated to the carotenoid esters phenotype. Consequently, breeding for carotenoid enhancement should then focus on Type 1 *XAT-7A1*, which is associated with high levels of carotenoid esterification, and can be assessed as for a single dominant gene strategy as *XAT-7A1* copies are inherited together. In this context, homozygous individuals from the F_2_ population constitute an excellent pre-breeding material for the transference of *XAT-7A1* to durum wheat elite varieties.

## Methods

### Plant material

A total of 156 accessions from a Spanish durum wheat landrace collection [26, 51] were used to test for the presence of the XAT candidate gene in durum wheat. This collection was originally provided by the National Centre for Plant Genetic Resources (CRF-INIA-CSIC) as described in [26]. Seedlings from the landraces BGE047520, BGE047535, BGE048494 and BGE047499 from the former collection were selected and grown in field following a completely randomized design with two replicates (blocks) for expression analyses (see below). BGE047520 and BGE047535 show diesters and monoesters in high proportion, BGE048494 monoesters in low concentration and BGE047499 no carotenoid esters in their grain carotenoid profiles.

In addition, an F_2_ population was developed by crossing the landrace BGE047535 as the maternal parent with the zero-ester commercial cultivar ‘Athoris’ (LG Seeds). The resulting F_1_ hybrids were checked with molecular markers to ensure true crosses and a F_2_ population of 120 individuals was obtained by selfing. This population was grown at field conditions at Finca Alameda del Obispo (Córdoba, Spain) using anti-weed nets and anti-bird structure for the determination of carotenoid content and profile in grain.

### DNA isolation

Genomic DNA was extracted using the CTAB method as described in [52] with the specifications described by [32] from young leaves from the parental lines and the BGE047535 × ‘Athoris’ F_2_ population. Genomic DNA from the landraces collection was isolated from young leaves following the same method in a previous work [53].

### Extraction of carotenoids and HPLC analysis

Carotenoids pigments were extracted from mature grains from the BGE047535 × ‘Athoris’ F_2_ population as previously described in [32]. The Spanish durum wheat landraces collection of 156 accessions had been characterized for grain carotenoid and xanthophyll esters profile in previous works [26, 51]. Carotenoid extraction and analysis was performed in duplicate under dimmed light to avoid carotenoid isomerization and photo-degradation. All the analyses were carried out on the same day as the extract was prepared. Carotenoids analysis was performed by HPLC as described in previous works [24]. Calibration curves, prepared with pure pigment standards, were used for carotenoid quantification. The concentration of (*Z*)-isomers of lutein was assessed by using the calibration curve for (*all-E*)-lutein. Lutein esters were determined as free lutein equivalents. All data were expressed as µg/g fresh weight (µg/g fw).

### Candidate gene amplification and sequence analysis

Genomic sequences of genes TraesCS7D02G094000, TraesCS4A02G397900, TRITD4Av1G231840 and TRITD4Av1G231510 were retrieved from Ensembl Plants and aligned using the multiple sequence alignment tool ClustalW [54]. Primers were designed in the conserved 5’ and 3’ regions by using the NCBI Primer-Blast tool [55]. Primer pair XAT_dw-Fw/ XAT_dw-Rv (Additional file 2) was tested in the Spanish durum wheat collection and successfully amplified a 1,564 bp length fragment in some of the accessions. PCR reactions were carried out with Velocity DNA Polymerase (Bioline, London, UK). The amplicons were cloned into pGEMT-Easy vector (Promega, Madison, WI) and transformed into competent *Escherichia coli* (DH5α) cells. Plasmids were purified using Zyppy^TM^ Plasmid Miniprep Kit (Zymo Research, CA, US) and used as template for sequencing (STAB VIDA, Portugal).

Sequence editing, alignment and assembly were performed with SeqMan Pro Lasergene Software v17 (DNAStar, WI, US). A minimum of 12 clones were sequenced from genotypes BGE047520 and BGE047535, and five from each of BGE048494, BGE047499 and ‘Athoris’. A minimum of three clones of XAT_dw-Fw/ XAT_dw-Rv amplicon was analyzed in accessions BGE047507, BGE047536, BGE012301, BGE045645, BGE047498, BGE047503, BGE048496, BGE045628, BGE045676, BGE048499.

The identity of the clones as GDSL esterase-lipase- like sequences was confirmed by BLASTn at NCBI. The coding sequences were predicted based on the exon-intron structure of TraesCS7D02G094000, and open reading frames were searched by using ORFfinder at NCBI (https://www.ncbi.nlm.nih.gov/orffinder). Signal peptide and cellular location of the expected proteins were predicted by SignalP 6.0 software [56].

### Genotyping, map construction and QTL analyses

A subset of 90 individuals from the BGE047535 × ‘Athoris’ F_2_ population was selected for map construction. Genotyping by sequencing analysis of the mapping population was performed by means of DArTSeq platform at Diversity Arrays Technology Pty Ltd (Canberra, Australia).

The genetic map was constructed using Joinmap ® v. 5.0 (Kyazma ®, The Netherlands). Only DArTSeq markers with a call rate of above 90% and with consistent segregation in the parental lines of the population were used for mapping. Markers deviating from mendelian segregation were excluded. A minimum LOD of 16.0 was used to assign markers to chromosomes. For each chromosome, several rounds of mapping were performed by excluding markers co-segregating in the same positions and using the EML algorithm (fastest). A final round of mapping was performed using the Regression Mapping algorithm and the Kosambi mapping function to get the final maps. Collinearity between genetic map and physical positions were inspected using CIRCOS [57] based on DArTSeq markers with a significant match to ‘Svevo’ genome after BLASTn alignment (E-value < 1.5 × 10−6).

Total carotenoid content and degree of lutein esterification were used for QTL analyses with MapQTL ® v. 6.0 (Kyazma ®, The Netherlands). The nonparametric Kruskal-Wallis test was used to identify marker-trait association in a first stage. After this, interval-mapping analyses were carried out [58, 59]. Finally, MQM mapping was performed [60–62]. The QTL significance (*p-*value) was calculated by using a permutation analysis (1,000 permutations) [63]. QTL figures were generated by using MapChart software v2.32 [64].

### Development of SNP markers

Two markers were designed for mapping purposes following a Tetra-Primer ARMS (amplification refractory mutation system) strategy for SNPs detection [65]. Primer1 web service (http://primer1.soton.ac.uk/primer1.html) was used for primer design. A SNP between BGE047535 and ‘Athoris’ XAT-like copies at position 1243 bp was scored in the mapping population with primers SNP_1243_C-Fw, SNP_1243_T-Rv, SNP_1243_out-Fw and SNP_1243_out-Rv (Additional file 2). A second SNP at position 1220 bp, polymorphic between BGE047535 copies, was scored with primers SNP_1220_A-Fw, SNP_1220_T-Rv, SNP_1220_out-Fw and SNP_1220_out-Rv (Additional file 2). PCR amplifications were performed following the manufacturer’s instructions with MyTaq^TM^ DNA polymerase (Bioline, London, UK) and resolved in agarose gels stained with Safeview^TM^ Nucleic Acid Stain (NBS biologicals, Ltd., Cambridgeshire, England).

### Expression analysis

Durum wheat landraces BGE047520, BGE047535, BGE048494 and BGE047499 were grown following a completely randomized design with two replicates at field conditions. Developing grains at 18 (Zadoks 77), 25 (Zadoks 83-85) and 30 (Zadoks 87) days post-anthesis (dpa) [66], which correspond to late milk, early-soft dough and hard dough, respectively, were used as samples. Two independent biological replicates were collected from each block and immediately frozen in liquid nitrogen at −80 °C. Four grains were randomly selected from each biological replicate for RNA extraction. Total RNA isolation was carried out in duplicate from whole grains using the TRIzol® Reagent (Invitrogen, CA, US), according to manufacturer’s instructions with minor modifications. cDNA was obtained as previously described [67].

Real-time qPCR reactions for expression analysis were carried out using SYBR® Green on an Applied Biosystems™ 7500 Real-Time PCR System (Applied Biosystems, CA, USA). Four biological replicates with two technical duplicates (which consisted in 1:4 dilutions of each sample), were used as templates. The primer pair qXAT_dw-Fw / qXAT_dw-Rv (Additional file 2) was designed in a highly conserved region from the end of exon four to the beginning of exon five comprising intron four. The following PCR conditions were used: 95°C for 10 min followed by 40 cycles at 95°C for 15 s and 60°C for 1 min. qPCR reactions were performed by using 5 μl of diluted cDNA (10 ng/μl), 5 μl of iTaqTM Universal SYBR® Green Supermix (Bio-Rad, CA, US), and a primer pair concentration of 0.22 μM each. Control samples with no template were included in the reactions.

LinRegPCR quantitative PCR data analysis program (version 11.0) [68] was used for determining PCR efficiency, using raw normalized fluorescence as input data. Expression for each sample (N0) was calculated using the equation N0 = 0.2/E^Cq^, being E the PCR efficiency for each primer and Cq the number of cycles needed to reach 0.2 arbitrary units of fluorescence.

Normalization was carried out by using the geometric mean of the reference genes ADP-RF(m), RLI(a) and CDC(a) [69]. Expression stability of these three genes and the normalization factors for each sample were assessed using GeNorm [70].

### Copy number variation (CNV) analysis

The primer pair CNV_XAT-Fw / CNV_XAT-Rv (Additional file 2) was designed in a highly conserved region in exon four for copy number variation analysis of XAT copies in genotypes BGE047520, BGE047535, BGE048494, BGE047499, BGE018318 and ‘Athoris’. Seven F_2_ individuals from the BGE047535 × ‘Athoris’ population were included. These individuals were assigned as BGE047535, ‘Athoris’ or heterozygous haplotypes based on the SNP_1220 and SNP_1243 genotyping and their phenotypic profile for carotenoid esterification (2, 2 and 3 individuals, respectively). Primer pairs TaFAD6 [71] and RLI(a) [69] were used as reference genes (Ref 1 and Ref 2, respectively).

The amplification profile was the same as described above for expression analysis. qPCR reactions were performed using 3,5 μl of DNA (6,25 ng/μl), 10 μl of iTaqTM Universal SYBR® Green Supermix (Bio-Rad, CA, US) and a primer pair concentration of 0.22 μM each in a final volume of 20 μl. Five biological replicates were used for landraces and ‘Athoris’, and three technical replicates for F_2_ individuals. PCR efficiency was determined as described above. The ratio between the target gene and each reference genes was calculated as described in by [72] including the efficiency correction for copy number estimation with the following formula:

$$Ratio=N \times \frac{{({E}_{\left(ref\right)})}^{{MCq}_{(ref)}}}{{({E}_{\left(target\right)})}^{M{Cq}_{(target)}}}$$

where N is the number of copies per haploid genome for each of the reference genes (*N* = 2, for RLI(a) and TaFAD6).

### Supplementary Information

**Additional file 1. **Search for XAT candidate genes in durum wheat genomes using *XAT-7D* (TraesCS7D02G094000) from common wheat as gene model.

**Additional file 2. **List of primers designed in this work.

**Additional file 3. **List of the 28 landraces showing positive amplification for XAT candidate gene. Total carotenoids and percentage of carotenoid esterification is shown. XAT-like Type is indicated for the sequenced accessions.

**Additional file 4. **Alignment of XAT-7A1 and XAT-7D proteins. The predicted active sites are shown with black triangles. Signal peptide in positions 1–22 is highlighted.

**Additional file 5. **Genetic map for the F_2_ population derived from the cross BGE047535 × 'Athoris' using DArTSeq markers.

**Additional file 6. **Carotenoid content and profile of the F_2_ population derived from the cross BGE047535 × 'Athoris'.

## Data Availability

All data generated and/ or analysed during the current study are included in this published article, in its supplementary information files or available from the corresponding author on reasonable request. *XAT-7A1*_Type1.1, *XAT-7A1*_Type1.2, *XAT-7A1*_Type1.3, *XAT-7A1*_Type1.4a, *XAT-7A1*_Type1.4b, *XAT-7A1*_Type2, *XAT-7A1*_Type3.1 and *XAT-7A1*_Type3.2 have been submitted to the GenBank database under the accession numbers: OR082954, OR082955, OR082956, OR082957, OR082958, OR082959, OR082960 and OR082961, respectively.

## Abbreviations

CNV: Copy number variation
Dpa: Days post anthesis
FU: Fluorescence units
GDSL: Motif consensus amino acid sequence of Gly, Asp, Ser, and Leu around the active site Ser
GELP: GDSL-type esterase/lipase protein
Indels: Insertion/ deletion
PAV: Presence/ absence variation
SNP: Single-nucleotide polymorphism
St: Developing stage
XAT: Xanthophyll acyl transferase
YPC: Yellow pigment content
