# Supplementary figures and images for: Towards carotenoid biofortification in wheat: identification of XAT-7A1, a multicopy tandem gene responsible for carotenoid esterification in durum wheat
Source: BMC Plant Biol. 2023 Sep 6;23:412. doi: 10.1186/s12870-023-04431-4 (PMC10481513; doi:10.1186/s12870-023-04431-4)

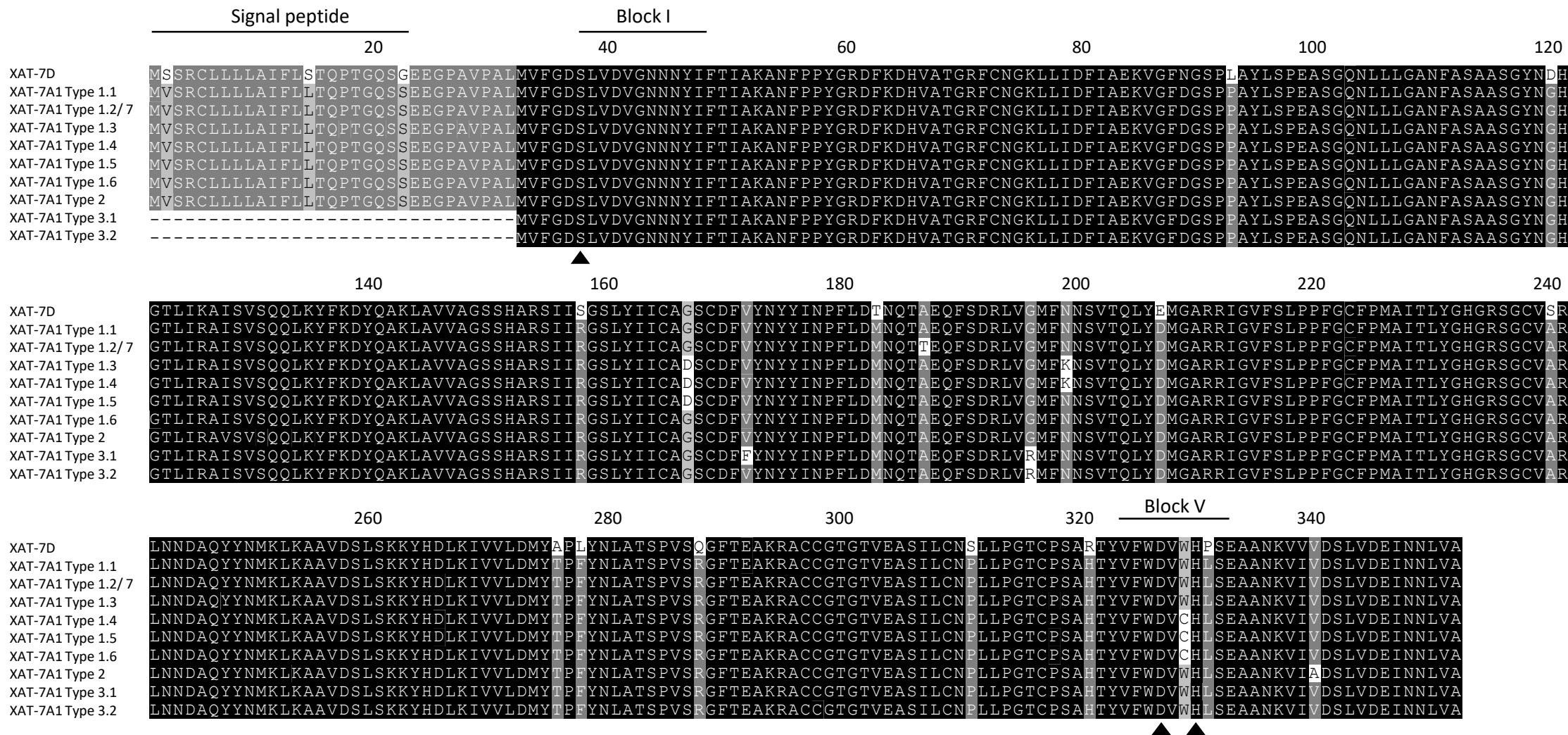

Supplement: Supplementary file 4 — Additional file 4. Alignment of XAT-7A1 and XAT-7D proteins. The predicted active sites are shown with black triangles. Signal peptide in positions 1–22 is highlighted. [file 12870_2023_4431_MOESM4_ESM.pdf]
